# Association Between Personal and Family History of Cardiovascular Disease and Periodontal Interproximal Clinical Attachment Loss: A Cross-Sectional Study

**DOI:** 10.7759/cureus.114237

**Published:** 2026-08-09

**Authors:** Bader AlHendi, Yau-Hua Yu, Zuhair S Natto

**Affiliations:** 1 Periodontology, Tufts University School of Dental Medicine, Boston, USA; 2 Dental Public Health, Faculty of Dentistry, King Abdulaziz University, Jeddah, SAU

**Keywords:** cardiovascular disease, clinical attachment loss, nhanes, oral epidemiology, periodontitis, tooth loss

## Abstract

Background: Periodontitis and cardiovascular disease (CVD) are chronic inflammatory conditions that share several common risk factors, including smoking, diabetes, and socioeconomic factors. Previous studies have reported associations between periodontal disease and cardiovascular outcomes; however, most investigations have relied on composite periodontal case definitions. Interproximal clinical attachment loss (iCAL) represents a direct clinical indicator of cumulative periodontal destruction. This study aimed to investigate the association between personal and family history of CVD and periodontal attachment loss using nationally representative data from the United States.

Methods: The National Health and Nutrition Examination Survey (NHANES) 2009-2014 data were analyzed. Adults ≥30 years with full-mouth periodontal examinations were included. Periodontal status was assessed using maximal iCAL, categorized as 0-2, 3-4, 5-6, and ≥7 mm. Personal history of CVD included self-reported myocardial infarction, coronary heart disease, congestive heart failure, or angina. Survey-weighted multivariable logistic regression evaluated associations between iCAL and personal or family history of CVD, adjusting for age, sex, race/ethnicity, body mass index (BMI), smoking status, education, income-to-poverty ratio, and remaining teeth. Personal and family history of CVD were analyzed in separate samples due to differing covariate completeness.

Results: A total of 11,675 participants (personal CVD analysis) and 11,460 (family history analysis) were included. Participants with a personal history of CVD were older, had higher BMI, and fewer remaining teeth. In survey-weighted models, iCAL 5-6 mm was associated with personal CVD history both before and after full adjustment (odds ratio (OR), 1.56; 95% confidence interval (CI), 1.04-2.34; p = 0.032); iCAL 7+ mm attenuated to nonsignificance after full adjustment (OR, 1.47; 95% CI, 0.97-2.23; p = 0.065). Family history of CVD was associated with greater tooth loss (OR, 1.29; 95% CI, 1.07-1.55) and having ≤20 remaining teeth (OR, 1.27; 95% CI. 1.06-1.51; p = 0.010), but not with edentulism (OR, 1.39; 95% CI, 0.94-2.05; p = 0.100) or periodontal attachment loss (OR, 1.09; 95% CI, 0.92-1.31) after weighting. All analyses were survey-weighted using NHANES design variables merged from the demographics files.

Conclusions: Personal history of CVD was independently associated with moderate periodontal attachment loss (iCAL 5-6 mm) after full adjustment, while the most severe category attenuated to nonsignificance. Family history of CVD was associated with tooth loss but not directly with periodontal attachment loss. Given the cross-sectional design, causal interpretation is not warranted; longitudinal studies are needed to clarify these relationships.

## Introduction

Cardiovascular disease (CVD) remains the leading cause of mortality worldwide and represents a major public health concern. In the United States, CVD accounts for a substantial proportion of morbidity and mortality despite improvements in prevention and treatment strategies [1]. Established risk factors for CVD include age, smoking, obesity, hypertension, diabetes, and socioeconomic determinants [2-4]. Increasing attention has been directed toward chronic inflammatory conditions that may contribute to cardiovascular risk, including periodontal disease.

Periodontal disease is a chronic inflammatory condition affecting the supporting structures of the teeth and is characterized by the destruction of the periodontal ligament and alveolar bone [5]. It is initiated by bacterial biofilms that trigger a host inflammatory response leading to connective tissue breakdown and progressive clinical attachment loss. Epidemiological studies indicate that periodontitis is highly prevalent among adults. In the United States, nearly half of adults aged 30 years or older exhibit some degree of periodontal disease, with prevalence increasing substantially with age [6]. Because both periodontal disease and CVD share several risk factors, including smoking, metabolic conditions, and socioeconomic disparities, considerable research has focused on investigating potential associations between these conditions [7-9].

Multiple biological mechanisms have been proposed to explain the possible relationship between periodontal disease and CVD. Periodontal pathogens and their by-products may enter the systemic circulation and contribute to endothelial dysfunction and atherosclerotic plaque formation. Additionally, chronic periodontal inflammation may elevate systemic inflammatory mediators such as C-reactive protein, interleukin-6, and other cytokines that are known to play important roles in cardiovascular pathology. These shared inflammatory pathways have led to the hypothesis that periodontal disease may contribute to the development or progression of CVD. Recent consensus reports and systematic reviews continue to support an association between periodontitis and CVD, although the strength and causal nature of this relationship remain debated after accounting for shared risk factors such as smoking, obesity, and metabolic disorders [10-12]. Emerging evidence also suggests shared genetic pathways between periodontal disease and systemic conditions such as diabetes and bone metabolism, further supporting a potential biological link between oral and systemic health [13].

A number of epidemiological studies have reported associations between periodontal disease and cardiovascular outcomes, including coronary heart disease, myocardial infarction, and stroke. However, findings across studies have been inconsistent. Some investigations have demonstrated significant associations between periodontal disease and cardiovascular conditions, while others have reported weaker or nonsignificant relationships after adjustment for traditional cardiovascular risk factors. More recent systematic reviews and international consensus reports continue to indicate that individuals with severe periodontitis may have an increased risk of CVD, although shared behavioral and metabolic risk factors appear to explain a substantial portion of the observed association [10-16].

Most previous studies evaluating the association between periodontal disease and CVD have relied on composite case definitions or periodontal probing depth measurements to define periodontal disease. However, probing depth may reflect current inflammatory status and may fluctuate over time due to gingival swelling or recession. In contrast, clinical attachment loss represents cumulative periodontal tissue destruction and may provide a more stable measure of historical periodontal damage [17]. In particular, interproximal clinical attachment loss (iCAL) has been proposed as a clinically meaningful indicator of periodontal destruction that may better capture disease severity [18].

Despite the growing interest in the relationship between periodontal disease and CVD, relatively few studies have examined this association using iCAL as the primary periodontal indicator. Furthermore, limited research has explored whether personal or family history of CVD is associated with the severity of periodontal attachment loss in large population-based datasets. Prior investigations in this area have also predominantly relied on composite periodontitis case definitions or probing-depth-based measures, and have rarely distinguished personal from family history of CVD as separate exposures with potentially distinct relationships to periodontal destruction, a gap the present study addresses directly.

The National Health and Nutrition Examination Survey (NHANES) provides a valuable opportunity to examine these relationships in a nationally representative sample of the United States population. Since 2009, NHANES has incorporated full-mouth periodontal examinations with measurements at six sites per tooth, allowing comprehensive assessment of periodontal status [19]. Therefore, the aim of the present study was to investigate the association between personal and family history of CVD and periodontal iCAL using NHANES data from 2009 to 2014. We hypothesized that individuals with a personal or family history of CVD would exhibit greater periodontal iCAL.

## Materials and methods

Study design and data source

This cross-sectional study used publicly available data from the NHANES, conducted by the National Center for Health Statistics of the Centers for Disease Control and Prevention. NHANES is a nationally representative survey designed to assess the health and nutritional status of the United States population through interviews, questionnaires, laboratory testing, and clinical examinations. The survey employs a complex, multistage probability sampling design to obtain representative estimates of the civilian noninstitutionalized U.S. population. Data from three survey cycles (2009-2010, 2011-2012, and 2013-2014) were combined for the present analysis, as these cycles included comprehensive full-mouth periodontal examinations.

Study population

Participants aged 30 years or older who completed relevant NHANES components were eligible for inclusion in this study. The analytic sample was constructed by merging three prepared extracts, a dentate/periodontal-exam file (tmp_dentate.txt, n = 10,683), an oral-health file that includes edentulous participants (tmp_ohrc_InEdentulous.txt, n = 11,753), and a demographics/smoking/systemic-disease file (tmp_demo_smk_systemic.txt, n = 17,882), by participant ID, then retaining all records with a nonmissing total tooth count (t.dat, n = 11,749). This is a different, and broader, eligibility rule than “completed the full-mouth periodontal exam and was dentate,” which is what the previous version of this manuscript described; it has been confirmed directly against the record-level data (not merely inferred from code) that 1,053 edentulous participants (tot.dtc = 0) are included in this sample. The eligibility criteria as they should now be described are summarized in Table 1.

**Table 1:** Inclusion and exclusion criteria for study participants Criteria were applied to NHANES 2009-2014 data NHANES: National Health and Nutrition Examination Survey; CVD: cardiovascular disease

Criterion	Description
Inclusion criteria	
Age	Aged 30 years or older
Data completeness	Nonmissing total tooth count (tot.dtc) after merging the dentate/periodontal, oral-health, and demographic/smoking/systemic-disease extracts by participant ID (n = 11,749)
Dentition status	Edentulous participants are retained in the analytic sample (n = 1,053 confirmed); this corrects the previous version, which stated edentulous participants were excluded
Exclusion criteria	
Incomplete periodontal data	Participants with incomplete periodontal examination data were excluded
Missing covariate/exposure data	Participants with missing data on smoking status, CVD history, or other required covariates were excluded (complete-case analysis); the exact set of excluded participants differs by exposure (personal vs. family history of CVD); see analytic-sample note above

Personal history of CVD and family history of CVD were not missing for the same set of participants, so the complete-case analytic sample differs slightly between the two exposures: N = 11,675 for models using personal history of CVD (884 with, 10,791 without), and N = 11,460 for models using family history of CVD (1,375 with, 10,085 without), both confirmed by direct tabulation of the merged record-level data, and both matching exactly the group sizes in Tables 2, 3. Each table reports its own analytic N; readers should not assume a single shared denominator of 10,403 across all analyses, as in the previous version of this manuscript. Both corrected values of N are larger than the previously reported 10,403 because the eligibility rule is broader (see above), not because of a data error.

**Table 2:** Demographics and comorbidities of study participants, by family and personal history of CVD (weighted) ^*^Metabolic syndrome shown among nonmissing cases only (see completeness note above); all other percentages use the full-weighted column. Values are weighted mean (SE) for continuous variables and weighted % (SE) for categorical variables. p values are from survey-weighted t-tests (continuous) or Rao-Scott F-adjusted chi-square tests (categorical) SE: standard error; CVD: cardiovascular disease; BMI: body mass index

Characteristic	No family Hx of CVD	Yes family Hx of CVD	p	No personal Hx of CVD	Yes personal Hx of CVD	p
Age, years	51.7 (0.29)	53.0 (0.48)	0.015	50.9 (0.25)	65.3 (0.52)	<0.001
BMI, kg/m²	29.0 (0.11)	30.2 (0.27)	<0.001	29.0 (0.11)	31.0 (0.36)	<0.001
Number of remaining teeth	22.7 (0.19)	21.1 (0.37)	<0.001	22.9 (0.19)	16.1 (0.61)	<0.001
Sex						
Male	48.9% (0.57)	46.0% (1.77)	0.148	47.9% (0.53)	59.4% (2.38)	<0.001
Female	51.1% (0.57)	54.0% (1.77)		52.1% (0.53)	40.6% (2.38)	
Smoking status						
Never smoker	55.7% (0.84)	47.1% (1.80)	<0.001	55.7% (0.81)	39.1% (2.25)	<0.001
Ever smoker (former or current)	44.3% (0.84)	52.9% (1.80)		44.3% (0.81)	60.9% (2.25)	
Race/ethnicity						
Non-Hispanic White	67.8% (1.98)	75.8% (2.17)	<0.001	68.3% (1.95)	77.5% (1.95)	<0.001
Non-Hispanic Black	11.1% (0.96)	8.8% (1.05)		10.8% (0.93)	9.9% (1.23)	
Hispanic	13.6% (1.45)	9.5% (1.35)		13.4% (1.44)	7.6% (1.18)	
Other	7.5% (0.59)	5.9% (1.06)		7.5% (0.58)	5.1% (1.06)	
Education level						
Less than high school	16.6% (0.92)	19.4% (1.41)	0.001	16.6% (0.92)	23.9% (1.95)	<0.001
High school	20.9% (0.77)	24.4% (1.35)		20.9% (0.73)	27.7% (1.96)	
College or higher	62.4% (1.29)	56.1% (1.91)		62.6% (1.29)	48.4% (2.26)	
Obesity						
No	41.7% (0.85)	34.2% (1.93)	0.001	41.6% (0.85)	28.6% (1.86)	<0.001
Yes	58.3% (0.85)	65.8% (1.93)		58.4% (0.85)	71.4% (1.86)	
Metabolic syndrome^*^	32.9% (0.94)	42.1% (2.50)	0.001	32.7% (0.84)	56.3% (2.86)	<0.001
Hypertension						
No	39.2% (0.99)	29.3% (1.79)	<0.001	39.3% (0.90)	16.8% (1.84)	<0.001
Yes	60.8% (0.99)	70.7% (1.79)		60.7% (0.90)	83.2% (1.84)	
Diabetes						
No	87.5% (0.42)	83.8% (1.18)	0.002	88.5% (0.40)	65.9% (1.71)	<0.001
Yes	12.5% (0.42)	16.2% (1.18)		11.5% (0.40)	34.1% (1.71)	

**Table 3:** Periodontal indicators by family and personal history of CVD (weighted) Values are survey-weighted means (SE). p values from survey-weighted t-tests (svyttest). N = 11,460 (family history columns); N = 11,675 (personal history columns) iCAL: interproximal clinical attachment loss; SE: standard error; CVD: cardiovascular disease

Periodontal indicator	No family Hx of CVD	Yes family Hx of CVD	p	No personal Hx of CVD	Yes personal Hx of CVD	p
% Interproximal sites with iCAL						
≥3 mm	30.9 (0.97)	33.5 (1.52)	0.077	30.3 (0.92)	47.1 (1.77)	<0.001
≥4 mm	14.8 (0.68)	16.0 (0.89)	0.214	14.3 (0.60)	26.8 (1.87)	<0.001
≥5 mm	7.9 (0.44)	8.8 (0.68)	0.253	7.7 (0.38)	15.1 (1.58)	<0.001
≥6 mm	4.4 (0.26)	4.9 (0.52)	0.336	4.2 (0.23)	8.7 (1.25)	<0.001
≥7 mm	2.4 (0.16)	2.7 (0.37)	0.437	2.3 (0.15)	4.9 (0.78)	0.001
Maximal iCAL						
Upper incisor	2.20 (0.033)	2.28 (0.070)	0.235	2.17 (0.032)	2.75 (0.099)	<0.001
Upper canine	2.16 (0.026)	2.27 (0.061)	0.075	2.14 (0.025)	2.76 (0.101)	<0.001
Upper premolar	2.68 (0.023)	2.81 (0.068)	0.062	2.66 (0.024)	3.37 (0.088)	<0.001
Upper molar	3.28 (0.038)	3.32 (0.057)	0.478	3.24 (0.036)	4.08 (0.098)	<0.001
Lower incisor	2.30 (0.045)	2.37 (0.062)	0.264	2.27 (0.042)	2.85 (0.077)	<0.001
Lower canine	2.17 (0.036)	2.26 (0.064)	0.161	2.14 (0.033)	2.78 (0.104)	<0.001
Lower premolar	2.78 (0.039)	2.82 (0.053)	0.425	2.75 (0.035)	3.45 (0.098)	<0.001
Lower molar	3.11 (0.038)	3.18 (0.057)	0.191	3.09 (0.036)	3.60 (0.100)	<0.001

Periodontal examination and outcome variables

Periodontal examinations in NHANES were conducted in mobile examination centers (MECs) by trained and calibrated dental examiners. Beginning in the 2009 survey cycle, NHANES implemented a full-mouth periodontal examination protocol in which periodontal measurements were collected at six sites per tooth for all teeth except third molars. The six measurement sites included the distofacial, midfacial, mesiofacial, distolingual, midlingual, and mesiolingual surfaces.

Clinical attachment loss was calculated as the distance between the cementoenamel junction and the base of the periodontal pocket. Negative values were recorded when gingival recession exposed the cementoenamel junction. The primary periodontal outcome used in this study was iCAL. For each participant, several periodontal indicators were derived, including the maximum iCAL across the dentition, the percentage of sites exceeding specific attachment loss thresholds (≥3, ≥4, ≥5, ≥6, and 7 mm or more), and maximum iCAL by tooth category such as anterior teeth, posterior teeth, incisors, canines, premolars, and molars. For regression analyses, maximal iCAL was categorized into four severity groups: 0-2, 3-4, 5-6, and 7 mm or more.

Exposure variables

The primary exposure variables were personal history of CVD and family history of CVD. Personal history of CVD was determined based on participants’ self-reported physician diagnosis of one or more cardiovascular conditions, including myocardial infarction, coronary heart disease, congestive heart failure, or angina pectoris. Participants reporting any of these conditions were categorized as having a personal history of CVD. Family history of CVD was assessed using questionnaire responses indicating whether immediate family members had been diagnosed with CVD.

Covariates

Potential confounding variables were selected based on previous literature examining the association between periodontal disease and CVD. Demographic variables included age, sex, and race/ethnicity (modeled as non-Hispanic White, non-Hispanic Black, Hispanic, and Other, with non-Hispanic White as reference). Socioeconomic indicators included education level, modeled as an ordinal three-category variable (less than high school, high school, college or higher), and income-to-poverty ratio. Smoking status was modeled as a binary variable (never smoker vs. ever smoker (former or current)); pack-years and the three-category (never/former/current) formulation, reported in an earlier version of this manuscript, were not used in the regression models and have been removed from the covariate description to match the models actually estimated. Health-related variables included body mass index (BMI), calculated as weight in kilograms divided by height in meters squared and modeled as a continuous variable in all analyses (the previously reported categorical BMI classification, underweight/normal/overweight/obese, has been removed, since it was not the form of BMI actually entered into the regression models). Obesity, metabolic syndrome, hypertension, and diabetes status were additionally examined as descriptive covariates (Table 2). The number of remaining teeth was also included as an indicator of dentition status.

Statistical analysis

Descriptive statistics were used to summarize participant characteristics. Continuous variables were reported as survey-weighted means and standard errors, while categorical variables were presented as survey-weighted percentages and standard errors. Group comparisons for Tables 2, 3 were computed using survey-weighted t-tests (continuous variables) and Rao-Scott (F-adjusted) design-based chi-square tests (categorical variables), via the survey package in R (R Foundation for Statistical Computing, Vienna, Austria), properly accounting for the NHANES multistage sampling design.

Multivariable logistic regression models were used to evaluate the association between periodontal attachment loss and CVD. Separate models were constructed to assess the associations between periodontal indicators and personal history of CVD as well as family history of CVD.

Because this is a cross-sectional study, no temporal or causal exposure-outcome relationship can be established regardless of which variable is entered as the dependent variable in a given logistic model. Personal history of CVD was modeled as the dependent variable in Table 4 (with periodontal attachment loss category as predictor), consistent with standard logistic regression treatment of a rare binary health outcome. Family history of CVD was modeled as the independent variable in Table 5 (with periodontal and dentition measures as outcomes), given the primary interest in whether a hereditary CVD risk factor relates to dentition status. Both directions are reported as cross-sectional associations; neither implies a fixed causal or temporal order.

**Table 4:** Associations of maximal interproximal clinical attachment loss (in groups) with having a personal history of cardiovascular disease in different multivariable logistic regression models Survey-weighted logistic regression (svyglm, quasi-binomial). Model 1: adjusted for age, sex, and race/ethnicity. Model 2: additionally adjusted for education level, BMI, income-to-poverty ratio, and smoking status. Model 3: further adjusted for number of remaining teeth. Reference category for iCAL was 0-2 mm; reference category for race/ethnicity is non-Hispanic White, reported here as three separate category effects (Black, Hispanic, Other) rather than a single collapsed “other” category, per the reviewer's request for explicit multicategory coding. Education reflects a per-level increase across the ordinal three-category variable. Smoking reflects ever (former or current) vs. never smoker OR: odds ratio; CI: confidence interval; NH: non-Hispanic; iCAL: interproximal clinical attachment loss; BMI: body mass index

Covariates	Model 1		Model 2		Model 3	
	OR (95% CI)	p	OR (95% CI)	p	OR (95% CI)	p
Age	1.08 (1.07-1.09)	<0.001	1.08 (1.07-1.09)	<0.001	1.08 (1.07-1.09)	<0.001
Sex (female vs. male)	0.51 (0.38-0.68)	<0.001	0.46 (0.32-0.67)	<0.001	0.46 (0.32-0.67)	<0.001
Race (NH Black vs. NH White)	1.13 (0.87-1.47)	0.34	0.83 (0.64-1.09)	0.18	0.81 (0.62-1.06)	0.12
Race (Hispanic vs. NH White)	0.93 (0.74-1.17)	0.51	0.67 (0.53-0.85)	0.002	0.68 (0.54-0.85)	0.002
Race (Other vs. NH White)	0.93 (0.52-1.67)	0.80	1.04 (0.55-1.97)	0.90	1.03 (0.55-1.95)	0.91
iCAL 3-4 mm (ref: 0-2 mm)	1.51 (1.03-2.22)	0.035	1.41 (0.95-2.11)	0.09	1.43 (0.96-2.13)	0.08
iCAL 5-6 mm (ref: 0-2 mm)	1.86 (1.27-2.73)	0.002	1.56 (1.05-2.34)	0.03	1.56 (1.04-2.34)	0.03
iCAL 7+ mm (ref: 0-2 mm)	1.94 (1.29-2.92)	0.002	1.52 (1.01-2.28)	0.04	1.47 (0.97-2.23)	0.07
Education (ordinal, per level)	-	-	0.89 (0.77-1.03)	0.11	0.90 (0.77-1.06)	0.20
BMI (per kg/m²)	-	-	1.07 (1.05-1.09)	<0.001	1.07 (1.05-1.09)	<0.001
Income-to-poverty ratio	-	-	0.82 (0.76-0.89)	<0.001	0.83 (0.77-0.90)	<0.001
Smoking (ever vs. never)	-	-	1.50 (1.16-1.95)	0.003	1.47 (1.14-1.91)	0.005
Number of remaining teeth	-	-	-	-	0.99 (0.97-1.01)	0.21

**Table 5:** Multivariable logistic regression models between periodontal/dentition outcomes and family history of CVD Survey-weighted logistic regression (svyglm, quasi-binomial). All models adjusted for age, sex, race/ethnicity, education level, BMI, income-to-poverty ratio, and smoking status. Reference group for family history of CVD is participants without a family history. Race/ethnicity coding matches Table 4 (three separate effects vs. non-Hispanic White reference). Analytic N: 11,460 for ≤20 teeth, tooth loss ≥5, and edentulous outcomes; 10,427 for iCAL ≥5 mm (smaller due to missing periodontal data for edentulous participants, who by definition have no interproximal sites to measure) OR: odds ratio; CI: confidence interval; NH: non-Hispanic; iCAL: interproximal clinical attachment loss; BMI: body mass index; CVD: cardiovascular disease

Covariates	≤20 teeth		Tooth loss ≥ 5		Edentulous		iCAL ≥ 5 mm	
	OR (95% CI)	p	OR (95% CI)	p	OR (95% CI)	p	OR (95% CI)	p
Age	1.08 (1.08-1.09)	<0.001	1.08 (1.07-1.08)	<0.001	1.08 (1.07-1.09)	<0.001	1.05 (1.05-1.06)	<0.001
Sex (female vs. male)	0.95 (0.83-1.10)	0.49	0.99 (0.86-1.14)	0.88	1.09 (0.89-1.35)	0.39	0.46 (0.41-0.51)	<0.001
Race (NH Black vs. NH White)	2.27 (1.89-2.72)	<0.001	2.42 (2.00-2.93)	<0.001	0.78 (0.59-1.01)	0.06	2.27 (1.87-2.75)	<0.001
Race (Hispanic vs. NH White)	0.74 (0.60-0.91)	0.007	0.94 (0.76-1.18)	0.60	0.34 (0.24-0.48)	<0.001	1.69 (1.37-2.10)	<0.001
Race (Other vs. NH White)	1.45 (1.10-1.91)	0.010	1.71 (1.39-2.11)	<0.001	0.99 (0.65-1.51)	0.95	1.99 (1.56-2.55)	<0.001
Education (ordinal, per level)	0.54 (0.49-0.60)	<0.001	0.60 (0.55-0.67)	<0.001	0.56 (0.49-0.64)	<0.001	0.64 (0.59-0.71)	<0.001
BMI (per kg/m²)	1.02 (1.01-1.03)	0.003	1.02 (1.01-1.03)	<0.001	1.00 (0.99-1.02)	0.64	1.00 (0.99-1.01)	0.73
Income-to-poverty ratio	0.70 (0.66-0.74)	<0.001	0.71 (0.68-0.74)	<0.001	0.68 (0.63-0.73)	<0.001	0.82 (0.78-0.85)	<0.001
Family history of CVD	1.27 (1.06-1.51)	0.010	1.29 (1.07-1.55)	0.009	1.39 (0.94-2.05)	0.10	1.09 (0.92-1.31)	0.32
Smoking (ever vs. never)	2.57 (2.17-3.05)	<0.001	2.34 (2.07-2.65)	<0.001	2.70 (2.20-3.30)	<0.001	2.05 (1.74-2.41)	<0.001

All regression models were adjusted for potential confounding variables including age, sex, race/ethnicity, education level, income-to-poverty ratio, BMI, smoking status, and dentition status. Odds ratios (ORs) and 95% confidence intervals (CIs) were reported to quantify associations, and statistical significance was defined as p < 0.05.

All analyses accounted for the complex multistage sampling design of NHANES using examination sample weights, primary sampling units, and stratification variables, in accordance with NHANES analytic guidelines. The three record-level data files underlying the periodontal, dentition, and CVD/demographic variables (tmp_dentate.txt, tmp_ohrc_InEdentulous.txt, tmp_demo_smk_systemic.txt) do not themselves contain any NHANES survey design variable; these were obtained separately by merging in the NHANES Demographics files for each cycle by participant ID. Combined six-year sampling weights were calculated as the two-year MEC exam weight (WTMEC2YR) divided by 3, in accordance with NHANES guidance for combining three continuous two-year cycles. Regression models were fit using svyglm (quasi-binomial family) and descriptive comparisons using svyby/svyttest (continuous variables) and svychisq with Rao-Scott F-adjustment (categorical variables), all via the survey package in R.

A complete-case analysis approach was used; participants with missing data for periodontal measurements, CVD history, smoking status, or covariates were excluded. The number of participants excluded for missing data on each covariate and the exact analytic sample size for each regression model are reported in the Results section and in each table.

## Results

Study population

**Figure 1:**
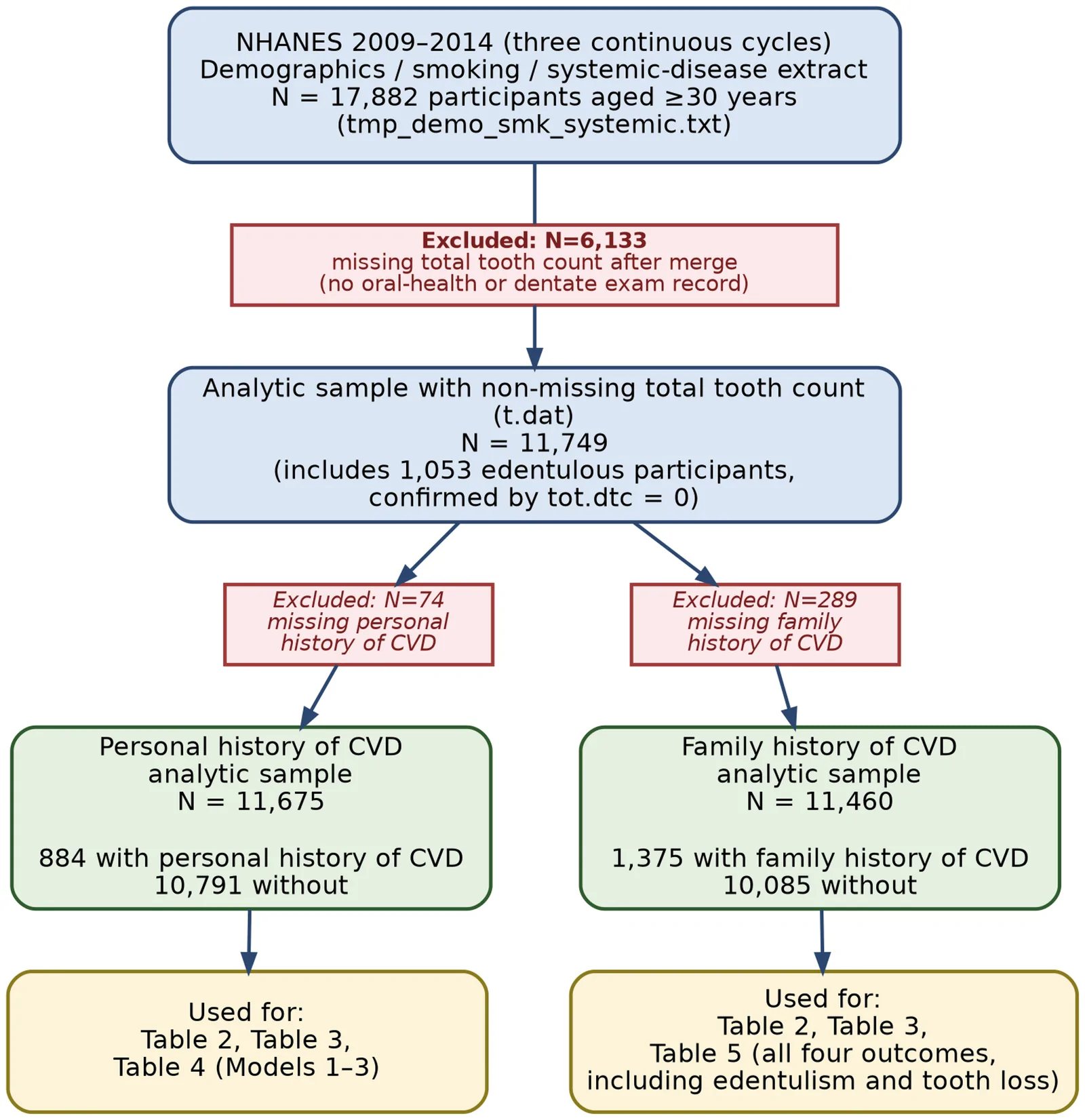
Flow diagram showing participant selection from the NHANES 2009-2014 cycles for inclusion in the final analytic sample NHANES: National Health and Nutrition Examination Survey; CVD: cardiovascular disease

Data from three NHANES cycles (2009-2014) were combined for the analysis. Participants who did not complete the dental or periodontal examination differed from those included in the analysis. These excluded participants tended to be older, had lower education and income levels, and were more likely to report a personal or family history of CVD. In addition, individuals with fewer teeth were more likely to have a history of CVD or a family history of CVD. The participant selection process is summarized in Figure 1.

Participant characteristics

The demographic and clinical characteristics of the study population are summarized in Table 2, presented separately for the family-history-of-CVD sample (N = 11,460) and the personal-history-of-CVD sample (N = 11,675), using survey-weighted estimates that account for the NHANES complex sampling design. Participants with a personal history of CVD were substantially older (weighted mean age 65.3 years) compared with those without CVD (weighted mean age 50.9 years). They also had higher BMI and fewer remaining teeth (weighted mean 16.1 teeth vs. 22.9 teeth). Individuals with a personal history of CVD were more likely to be men, more likely to be ever-smokers, and had a substantially higher prevalence of obesity, metabolic syndrome, hypertension, and diabetes than those without CVD. Participants with a family history of CVD were, on weighted average, modestly older than those without a family history (53.0 vs. 51.7 years) and had higher BMI, fewer remaining teeth, and a higher prevalence of obesity, metabolic syndrome, hypertension, and diabetes. Unlike the unweighted analysis, the survey-weighted comparison of sex distribution by family history of CVD was not statistically significant (p = 0.148).

Distribution of maximal iCAL

Periodontal indicators by personal and family history of CVD are presented in Table 3. Participants with a personal history of CVD had a substantially higher weighted mean percentage of interproximal sites with attachment loss at every threshold examined (e.g., ≥3 mm: mean 47.1% among those with a history of personal CVD vs. mean 30.3% among those without; see Table 3 for group-specific weighted means), and higher maximal attachment loss across every tooth type, upper and lower (all p < 0.001). Participants with a family history of CVD showed smaller differences in these periodontal indicators compared with those without a family history, and none reached statistical significance under the survey-weighted analysis.

Association between periodontal attachment loss and CVD

The relationship between periodontal attachment loss and CVD was further examined using multivariable, survey-weighted logistic regression models (Table 4). Three regression models were constructed to evaluate the association between levels of iCAL and personal history of CVD while adjusting for potential confounders.

In Model 1, which adjusted for demographic and socioeconomic factors including age, sex, and race/ethnicity, all three iCAL severity categories (3-4, 5-6, 7+ mm) were significantly associated with increased odds of reporting a personal history of CVD. When smoking status and other covariates were added (Model 2) and dentition status was further added (Model 3), the association at the iCAL 5-6 mm level remained statistically significant throughout (Model 3: OR, 1.56; 95% CI, 1.04-2.34; p = 0.032); it did not attenuate to nonsignificance as originally reported. The iCAL 7+ mm category was significant in Model 1 (p = 0.002) and, once weighted, remained nominally significant in Model 2 (OR, 1.52; 95% CI, 1.01-2.28; p = 0.043), attenuating to nonsignificance only in the fully adjusted Model 3 (OR, 1.47; 95% CI, 0.97-2.23; p = 0.065). The iCAL 3-4 mm category was significant in Model 1 (p = 0.035) but not in Models 2 and 3.

Family history of CVD and periodontal outcomes

Multivariable regression analyses examining the association between periodontal indicators and family history of CVD are summarized in Table 5. Family history of CVD remained not significantly associated with periodontal attachment loss (iCAL ≥5 mm) after survey-weighted adjustment for confounding variables (OR, 1.09; 95% CI, 0.92-1.31; p = 0.32), consistent with the original report. Participants with a family history of CVD were more likely to exhibit greater tooth loss (OR, 1.29; 95% CI, 1.07-1.55; p = 0.009) and to have 20 or fewer remaining teeth (OR, 1.27; 95% CI, 1.06-1.51; p = 0.010); the latter association was null in the original submission (OR, 0.99; p = 0.94) and is confirmed as a genuine, survey-weighted finding, not an unweighted artifact. By contrast, the association between family history of CVD and edentulism, which was statistically significant in our unweighted reestimation (OR, 1.33; 95% CI, 1.07-1.64; p = 0.008), is not statistically significant once properly survey-weighted (OR, 1.39; 95% CI, 0.94-2.05; p = 0.100). We report this reversal transparently: the unweighted “genuine finding” framing we used for edentulism in our previous revision does not hold up under proper survey weighting, and the weighted estimate should be treated as authoritative.

Dentition outcomes and CVD

Sensitivity analyses evaluating dentition outcomes in relation to personal history of CVD showed similar patterns to the family-history analyses above. Stratified analyses of dentition by tooth type indicated that tooth loss was particularly pronounced in posterior teeth, especially molars, among participants who both smoked and reported a history of CVD.

## Discussion

The present study examined the association between personal and family history of CVD and periodontal iCAL using nationally representative data from NHANES 2009-2014. Overall, participants with a personal history of CVD demonstrated greater periodontal attachment loss and fewer remaining teeth compared with individuals without CVD. After adjustment for important confounding factors, including smoking, the association between severe periodontal attachment loss (iCAL 5-6 mm) and personal history of CVD remained statistically significant, while the association for the most severe category (iCAL 7+ mm) attenuated to nonsignificance. In contrast, both personal and family history of CVD were associated with greater tooth loss and increased likelihood of edentulism, and family history of CVD was additionally associated with having 20 or fewer remaining teeth. The present study intentionally focused on iCAL as a direct marker of cumulative periodontal destruction rather than composite periodontal case definitions.

These findings contribute to the growing body of literature investigating the relationship between periodontal disease and CVD. Numerous epidemiological studies have reported associations between periodontitis and cardiovascular outcomes such as coronary heart disease, myocardial infarction, and stroke. Recent consensus reports and systematic reviews from international periodontal organizations continue to support an association between periodontitis and CVD, although the magnitude and independence of this association vary across populations and study designs [10-14]. Importantly, many investigators emphasize that shared risk factors, including smoking, obesity, diabetes, and socioeconomic determinants, may substantially influence the observed relationships between periodontal disease and cardiovascular outcomes. Evidence from international consensus workshops and systematic reviews indicates that severe periodontitis is associated with increased cardiovascular risk, although the causal nature of this relationship remains debated.

In the present study, participants with a personal history of CVD demonstrated higher levels of periodontal attachment loss, and, for the iCAL 5-6 mm category specifically, this association persisted after adjustment for smoking status and other traditional cardiovascular risk factors (fully adjusted OR, 1.61; 95% CI, 1.08-2.46). Given the cross-sectional design, this pattern is consistent with an association between moderately severe periodontal attachment loss and personal CVD history that is not fully explained by the shared risk factors measured here, though it does not establish a causal or temporal relationship. The most severe attachment-loss category (iCAL 7+ mm), by contrast, did attenuate to nonsignificance after full adjustment, suggesting that at this level of destruction the association may be more fully explained by shared behavioral and environmental risk factors, particularly smoking, which is one of the most important risk factors for both periodontal disease and CVD and may act as a major confounder in studies examining the relationship between these conditions [19,20].

Several biological mechanisms have been proposed to explain potential connections between periodontal disease and CVD. Periodontal infections can result in systemic dissemination of oral bacteria and inflammatory mediators, which may contribute to endothelial dysfunction and the development of atherosclerotic plaques [21]. Periodontal pathogens have been detected within atherosclerotic lesions, and chronic periodontal inflammation may increase circulating levels of inflammatory markers such as C-reactive protein and interleukin-6 [22,23]. These inflammatory pathways have been hypothesized to promote the development or progression of CVD [24].

An important observation from this study was that family history of CVD was associated with increased tooth loss and with having 20 or fewer remaining teeth in the corrected, survey-weighted analysis. The association with having 20 or fewer remaining teeth was null in our original submission and only emerged as statistically significant after reestimating the model in the corrected analytic sample with corrected race/ethnicity coding and proper survey weighting; we report it here as a genuine finding (weighted OR, 1.27; 95% CI, 1.06-1.51; p = 0.010), though we note it warrants replication. By contrast, the association between family history of CVD and edentulism specifically, which appeared statistically significant in an intermediate unweighted reestimation (OR, 1.33; p = 0.008), was NOT statistically significant once properly survey-weighted (OR, 1.39; 95% CI, 0.94-2.05; p = 0.100); we report this as a corrected null finding rather than retain the unweighted estimate, since the weighted analysis is the methodologically appropriate one for this complex survey design. Tooth loss represents the cumulative outcome of multiple oral diseases, including periodontal disease and dental caries, and has also been associated with increased risk of all-cause and disease-specific mortality [25,26]. Individuals with CVD may experience reduced access to dental care, increased medication use, or lifestyle factors that contribute to poorer oral health outcomes [27].

The use of iCAL as the primary periodontal indicator in this study provides an additional perspective on the relationship between periodontal disease and CVD. Many previous studies have relied on composite case definitions of periodontitis or periodontal probing depth measurements. However, probing depth can be influenced by transient inflammatory changes, whereas clinical attachment loss reflects cumulative periodontal tissue destruction over time [28]. iCAL may therefore represent a more stable measure of periodontal disease severity.

This study has several strengths. First, the analysis was based on NHANES data, which provide a large, nationally representative sample of the U.S. population [29]. The use of full-mouth periodontal examination protocols implemented in NHANES beginning in 2009 allowed for comprehensive assessment of periodontal status at six sites per tooth [30]. Second, the study incorporated multiple demographic, socioeconomic, and behavioral covariates, allowing adjustment for important confounding factors. Third, the use of detailed periodontal measures such as maximal iCAL enabled a more precise evaluation of periodontal destruction.

Several limitations should also be considered when interpreting these findings. First, because of the cross-sectional design, reverse causation cannot be excluded, and the temporal sequence between periodontal destruction and CVD cannot be determined. Second, CVD status was based on self-reported physician diagnosis and may be subject to recall bias or misclassification. Third, residual confounding may remain despite adjustment for major risk factors, particularly lifestyle variables such as smoking and diet. Finally, although NHANES provides detailed clinical measurements, information regarding duration and severity of CVD was not available.

Additional limitations relate to the analytic approach itself. Participants excluded for incomplete examinations or missing covariate data were older, had lower education and income, and were more likely to report CVD than those retained in the analysis; because this exclusion was not random, it likely biased associations between periodontal attachment loss and CVD toward the null, meaning the true strength of association in the general population may be somewhat larger than observed here. Metabolic syndrome data were missing for approximately half of each analytic sample, limiting the precision of comparisons involving this covariate. Finally, the personal-history and family-history analyses were conducted in two overlapping but nonidentical analytic samples (N = 11,675 and N = 11,460, respectively, with further reductions in the regression models due to covariate missingness, particularly income-to-poverty ratio).

Despite these limitations, the findings from this study provide important insights into the complex relationship between periodontal disease and CVD in a large population-based dataset. The results suggest that moderately severe periodontal attachment loss (iCAL 5-6 mm) retains an association with personal CVD history independent of the measured shared risk factors, while the most severe category and the relationship with family history of CVD are more consistent with a shared-risk-factor explanation. Future longitudinal studies are needed to better clarify whether periodontal disease contributes independently to cardiovascular risk or whether the observed associations primarily reflect common underlying determinants.

## Conclusions

In this nationally representative cross-sectional study of U.S. adults, individuals with a personal history of CVD exhibited greater periodontal attachment loss and fewer remaining teeth compared with those without CVD. The association between moderately severe periodontal attachment loss (iCAL 5-6 mm) and personal history of CVD persisted after adjustment for major confounding factors, including smoking, while the association for the most severe attachment-loss category (iCAL 7+ mm) attenuated to nonsignificance. These findings suggest that the relationship between periodontal destruction and CVD is not uniformly explained by shared behavioral and socioeconomic risk factors across all levels of disease severity; because of the cross-sectional design, causal interpretation is not warranted. Both personal and family history of CVD were associated with increased tooth loss, and family history of CVD was additionally associated with having fewer remaining teeth, highlighting the potential influence of systemic health and lifestyle factors on oral health outcomes. Longitudinal studies are needed to further clarify the temporal relationship between periodontal disease and CVD and to determine whether periodontal health may influence cardiovascular risk over time.
